# Direct Estimates of the Genomic Contributions to Blood Pressure Heritability within a Population-Based Cohort (ARIC)

**DOI:** 10.1371/journal.pone.0133031

**Published:** 2015-07-10

**Authors:** Elias Salfati, Alanna C. Morrison, Eric Boerwinkle, Aravinda Chakravarti

**Affiliations:** 1 Center for Complex Disease Genomics, McKusick—Nathans Institute of Genetic Medicine, Johns Hopkins University School of Medicine, Baltimore, MD, 21205, United States of America; 2 Université Paris Descartes, Sorbonne Paris Cite, 75005, Paris, France; 3 Human Genetics Center, University of Texas Health Science Center, Houston, TX, 77030, United States of America; Medical University Hamburg, University Heart Center, GERMANY

## Abstract

Blood pressure (BP) is a heritable trait with multiple environmental and genetic contributions, with current heritability estimates from twin and family studies being ~ 40%. Here, we use genome-wide polymorphism data from the Atherosclerosis Risk in Communities (ARIC) study to estimate BP heritability from genomic relatedness among cohort members. We utilized data on 6,365,596 and 9,578,528 genotyped and imputed common single nucleotide polymorphisms (SNPs), in 8,901 European ancestry (EA) and 2,860 African Ancestry (AA) ARIC participants, respectively, and a mixed linear model for analyses, to make four observations. First, for BP measurements, the heritability is ~20%/~50% and ~27%/~39% for systolic (SBP)/diastolic (DBP) blood pressure in European and African ancestry individuals, respectively, consistent with prior studies. Second, common variants with allele frequency >10% recapitulate most of the BP heritability in these data. Third, the vast majority of BP heritability varies by chromosome, depending on its length, and is largely concentrated in noncoding genomic regions annotated as DNaseI hypersensitive sites (DHSs). Fourth, the majority of this heritability arises from loci not harboring currently known cardiovascular and renal genes. Recent meta-analyses of large-scale genome-wide association studies (GWASs) and admixture mapping have identified ~50 loci associated with BP and hypertension (HTN), and yet they account for only a small fraction (~2%) of the heritability.

## Introduction

Blood pressure (BP) is an established risk factor for multiple cardiovascular, stroke and renal disease and, worldwide, about one-tenth of adult global death is attributable to high BP or essential hypertension[1]. BP, studied through systolic (SBP) or diastolic (DBP) brachial artery measures or clinically defined HTN, is a complex, polygenic trait influenced by many environmental factors[2]. BP heritability is moderate, and ~40% across studies[3], and has classically been estimated from twin and family studies. Molecular genetic analyses of BP have been challenging with the exception of Mendelian hypo- and hypertension syndromes that show large BP variation in individuals harboring loss- or gain-of-function mutations in numerous renal genes[4]. These latter studies convincingly demonstrate that renal salt-water homeostasis is key to maintaining blood pressure control. Several environmental factors that influence BP levels, such as alcohol consumption, dietary salt-intake, physical activity and stress, are also known but their biochemical paths of action remain poorly described and understood. Consequently, the identification of genes that influence inter-individual variation in BP remains a key and important approach for the discovery of new etiological pathways in BP regulation.

In recent years, technical and analytic genomic advancements have made it feasible to conduct a comprehensive search for genes and regulatory loci underlying a trait of interest. To date, large-scale genome-wide association studies (GWAS), and other genome-wide analyses, have identified ~50 single nucleotide polymorphisms (SNPs) associated with genetic risk factors at high confidence (*P* < 5 × 10^-8^), and contributing to inter-individual variation in BP [5–14]. The vast majority of these associated SNPs is common in the general population (minor allele frequency, MAF>10%), and has been discovered to have small allelic effects (<0.05σ, where σ^2^ is the residual phenotype variance); collectively, these loci explain only a small (<5%) fraction of the phenotypic variance (heritability) [15]. This substantial gap between the expected and identified heritability has led to a great deal of speculation as to the causes of this “missing” heritability, mostly focusing on our failure to identify genetic effects of rare variants and copy number polymorphisms[16,17].

Nevertheless, before entertaining new genetic hypotheses for complex trait architecture it is first necessary to ask what is the total contribution of all *common genetic variation* to BP heritability? The typical approach to providing this answer has been through summing the contributions of individual SNPs showing genome-wide significant associations. This approach leads to a severe underestimate since GWASs suffer from a high false negative rate in its attempt to control the false positive rate. This false negative rate arises from the majority of genetic effects being too small to reach statistical significance and incomplete linkage disequilibrium between genotyped markers and causal variants.

Newer statistical methods allow a robust answer to this question by *estimating* a trait’s residual variance explained by all common SNPs taken together and by considering them as random effects in a mixed linear model[18,19]. Indeed, these analyses can be conducted on all genomic polymorphisms or those restricted to pre-identified subgroups, such as individual chromosomes, allele frequency class or functional annotation, to assess the relative contributions from these subgroups. The first of these analyses, by Visscher and colleagues, demonstrated that some complex traits arise largely from allelic effects of common (MAF>10%) variants [19–23]. In this study, we use their approach to ask: is inter-individual BP variation mostly due to polymorphic additive genetic factors? We also investigate the proportion of inter-individual BP variation that can be captured by common SNPs as a function of their chromosomal location (size), MAF of genotyped variants, broad functional class (promoter, DNaseI hypersensitive site (DHS), UTR, coding, intronic and intergenic; cardiovascular, renal or other genes), and by SNPs enriched for trait candidates (GWAS BP loci; Cardio-Metabochip SNPs). Finally, we also used longitudinal phenotype data, and assessed the effect of long-term average (LTA) BP, to detect additional genetic variance potentially by reducing measurement error [24]. The conclusions from these analyses demonstrate that the majority (>50%) of SBP and DBP heritability is from common genetic variation almost exclusively in non-coding (intronic and intergenic) DNA that is DNaseI hypersensitive and likely cis-regulatory. We also made the intriguing observation that the expected cardiovascular- and renal-related genes demonstrate some enrichment for AA subjects but have virtually no effect in EA participants. This implies considerable etiological differences in EA and AA BP effects. Our studies suggest an emerging these in complex trait genomics that identifying the causal factors for BP require more than larger GWAS studies but require an understanding of the cis-regulatory architecture of the human genome.

## Results

The BP distributions of the 8,901 EA and 2,860 AA individuals are not statistically different from the 6,914 unrelated-EA and 1,737 unrelated-AA, respectively, suggesting that the use of either set would be representative of the population’s BP features (Table 1). Relatedness between participants using genotyped only and genotyped and imputed SNPs followed normal distributions with mean -0.00015 (standard deviation, s.d. = 0.0044) and -0.00014 (s.d. = 0.0043), respectively, and showed trivial differences.

**Table 1:** Summary statistics of ARIC European ancestry (EA) and African Ancestry (AA) subjects used.

	European ancestry (EA) ARIC subjects					African ancestry (AA) ARIC subjects				
**Trait**	**N**	**Mean**	**SE**	**Min**	**Max**	**N**	**Mean**	**SE**	**Min**	**Max**
***SBP***	8901	121.29	19.44	61	221	2860	134.72	23.49	73	257
***DBP***	8901	73.54	11.52	12	139	2860	84.01	13.7	34	152
***AGE***	8901	54.27	5.7	44	66	2860	24.32	5.76	44	66
***BMI***	8901	26.97	4.83	14.38	56.26	2860	25.42	6.06	14.2	59.33
**Trait**	**Males**	**Mean**	**SE**	**Min**	**Max**	**Males**	**Mean**	**SE**	**Min**	**Max**
***SBP***	4197	122.99	18.28	61	203	1060	135.52	23.14	88	241
***DBP***	4197	75.59	11.37	12	130	1060	86.29	14.19	51	149
***AGE***	4197	54.67	5.7	44	66	1060	53.71	5.94	44	66
***BMI***	4197	27.46	3.96	17.21	56.26	1060	28.01	4.84	15.46	54.4
**Trait**	**Females**	**Mean**	**SE**	**Min**	**Max**	**Females**	**Mean**	**SE**	**Min**	**Max**
***SBP***	4704	119.76	20.3	72	221	1800	134.24	23.69	73	257
***DBP***	4704	71.71	11.34	27	139	1800	82.64	13.35	34	152
***AGE***	4704	53.9	5.67	44	66	1800	53.19	5.64	44	65
***BMI***	4704	26.54	5.46	14.38	55.2	1800	30.69	6.47	14.2	59.33

Consequently, for the remaining analyses we used the set of 8,901 EA and 2,860 AA individuals to maximize the available sample sizes.

The phenotypic variance explained by considering all imputed and genotyped SNPs for first visit measurement of blood pressure (V1) was 0.20 in EA (se = 0.03, *P* = 3.2x10^-10^) and 0.49 in AA (se = 0.13, *P* = 7.9x10^-4^) for SBP, and 0.27 in EA (se = 0.03, *P* = 5.2 x10^-11^) and 0.37 in AA (se = 0.12, *P* = 3.9 x10^-5^) for DBP: these were all highly significant estimates (Tables 2 and 3). We also estimated these same quantities but by using the genotyped SNPs only, namely 656,362 SNPs in EA and 772,638 SNPs in AA. We obtained, for first visit measurement (V1), a heritability of 0.25 in EA (se = 0.05, *P* = 2x10^-8^) and 0.45 in AA (se = 0.12, *P* = 1.1x10^-5^) for SBP, and 0.31 in EA (se = 0.05, *P* = 2 x10^-11^) and 0.29 in AA (se = 0.11, *P* = 7 x10^-5^) for DBP: these were also highly significant estimates. Not observing any major differences between these sets of estimates we used all imputed and genotyped SNPs in subsequent analyses since they represent the largest numbers of markers available.

**Table 2:** Proportion of the genetic variance explained by chromosome (joint) and the whole genome (combined) using 8,901 EA and 2,860 AA individuals. The trait analyzed is first-visit SBP and DBP and long-term average (LTA) SBP and DBP respectively using genotyped and imputed SNPs. The heritability (h^2^), its standard error (se) and significance value (P) are shown.

		SBP		DBP	
**EA**		V1 (N = 8,901)	LTA (N = 8,474)	V1 (N = 8,901)	LTA (N = 8,474)
**Chr**	**L_C_ (Mb)**	**h^2^ ± *s*.*e***.	**h^2^ ± *s*.*e***.	**h^2^ ± *s*.*e***.	**h^2^ ± *s*.*e***.
**Joint**	2,881.03	0.206	0.235	0.275	0.248
**Combined**		0.20 ± 0.035	0.23 ± 0.037	0.26 ± 0.035	0.23 ± 0.038
***P***		3.16x10^-10^	1.32x10^-10^	5.2x10^-11^	8.37x10^-11^
**AA**		V1 (N = 2,860)	LTA (N = 2,749)	V1 (N = 2,860)	LTA (N = 2,749)
**Joint**	2,881.03	0.504	0.5737	0.39	0.512
**Combined**		0.49 ± 0.126	0.57 ± 0.131	0.37 ± 0.122	0.55 ± 0.132
***P***		7.9x10^-6^	7.78x10^-7^	3.9x10^-5^	3.2x10^-7^

**Table 3:** Proportion of the genetic variance explained as a function of minor allele frequency (MAF) class using 8,901 EA and 2,860 AA individuals. The traits analyzed are first-visit SBP and DBP respectively for genotyped and imputed SNPs. The heritability (h^2^), its standard error (se) and significance value (P) are shown.

EA	Visit 1	
TRAIT	SBP	DBP
MAF (# SNPs)	*h* ^*2*^ *± s*.*e*.	*h* ^*2*^ *± s*.*e*.
0–0.1 (2,626,706)	0.025 ± 0.029	0.068 ± 0.031
0.1–0.2 (1,271,001)	0.056 ± 0.024	0.058 ± 0.025
0.2–0.3 (931,341)	0.046 ± 0.023	0.067 ± 0.025
0.3–0.4 (801,717)	0.028 ± 0.023	0.049 ± 0.023
0.4–0.5 (734,831)	0.034 ± 0.020	0.016 ± 0.019
**Total**	0.189 ± 0.035	0.257 ± 0.035
**Combined**	0.20 ± 0.035	0.26 ± 0.034
**P**	3.16x10^-10^	5.24x10^-11^
**AA**		
0–0.1 (6,119,379)	0.240 ± 0.156	0.104 ± 0.152
0.1–0.2 (1,464,651)	0.023 ± 0.111	0.073 ± 0.110
0.2–0.3 (843,903)	0.074 ± 0.095	0.101 ± 0.097
0.3–0.4 (623,815)	0.023 ± 0.084	0.000 ± 0.083
0.4–0.5 (526,779)	0.116 ± 0.074	0.063 ± 0.073
**Total**	0.478 ± 0.126	0.341 ± 0.128
**Combined**	0.49 ± 0.126	0.37 ± 0.122
**P**	7.94x10^-6^	3.94x10^-5^

**Fig 1:**
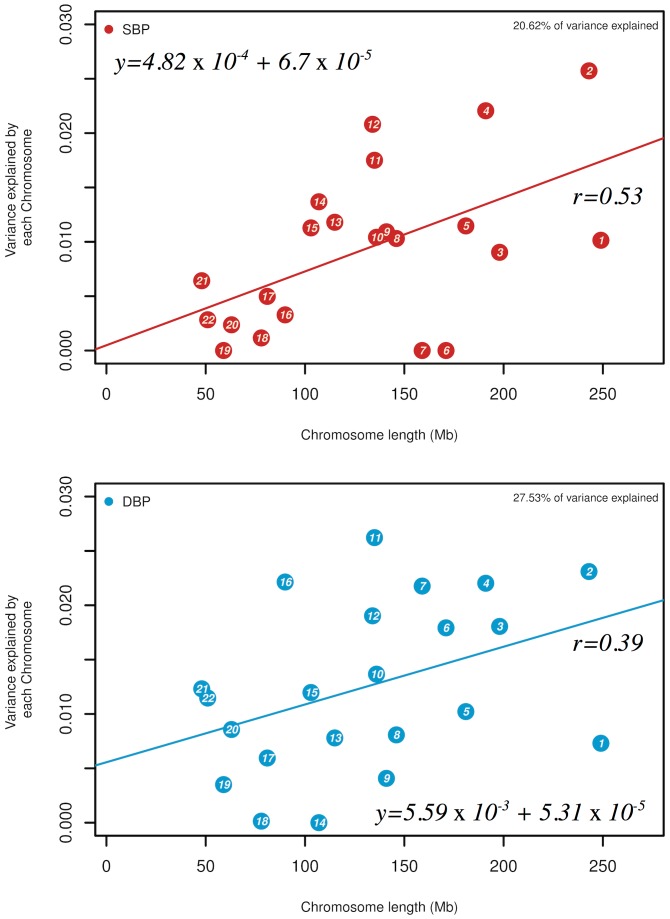
**(a)** Estimates of the variance explained by SNPs by chromosome (*h* ^*2*^_*c*_) for SBP (red) and DBP (blue) by joint analysis of 8,901 EA individuals. The trait analyzed is first-visit and long-term average SBP and DBP respectively with analyses using genotyped and imputed SNPs, **(b)** Legend as in **(a)** but for 2,860 AA individuals.

The total phenotypic variance explained can be apportioned by individual chromosomes and clearly demonstrated that although there is a general and significant correlation between chromosome length and variance explained (SBP r_*cor*_ = 0.53 (EA)/0.54 (AA); DBP r_*cor*_ = 0.39 (EA)/0.35 (AA)), individual chromosomes differed considerably in their contributions to SBP and DBP variation (Fig 1a and 1b). Nevertheless, there is high, but not absolute, concordance between the variances explained for both SBP and DBP by each chromosome. In EA the highest proportion of genetic variance captured by chromosomes for SBP is from three chromosomes: chromosomes 2 (*h* ^*2*^~2.6±1.1%), 4 (*h* ^*2*^~2.2±1.0%) and 12 (*h* ^*2*^~2.1±1.0%). Likewise, in AA the three chromosomes that account for the largest fraction of genetic variance for SBP were chromosomes 2 (*h* ^*2*^~13.0±5.0%), 5 (*h* ^*2*^~7.0±4.0%) and 11 (*h* ^*2*^~6.4±3.0%), capturing nearly half of the genetic variance. With respect to DBP, the most prominent contributions of genetic variation in EA were from chromosome: chromosomes 2 (*h* ^*2*^~2.3±1.1%), 4 (*h* ^*2*^~2.2±1.0%) and 11 (*h* ^*2*^~2.6±1.0%); whereas for AA, the three chromosomes that accounted for the highest variances, were chromosomes 2 (*h* ^*2*^~4.2±4.0%), 5 (*h* ^*2*^~8.7±4.5%) and 11 (*h* ^*2*^~5.1±3.0%), tagging over 40% of the genetic variance. These results support the notion that well over 50% of the BP heritability can be explained using existing genetic markers.

Blood pressure is a naturally varying phenotype so that increasing its precision may lead to improved genetic inferences. Thus, we assessed whether using the BP measurements from multiple (2–4) visits across time, as a Long Term Average (LTA), would lead to different conclusions by reducing measurement error [25,26]. We used the same set of genotyped plus imputed SNPs and analyzed SBP and DBP LTA heritability within ARIC (Table 2). The proportion of genetic variance captured by chromosome for SBP showed a high correlation with first visit values (EA: *r*_*cor*_ ~ 0.75, *P* = 8.84 x10^-5^; AA: *r*_*cor*_ ~ 0.78, *P* = 1.28 x10^-5^) with greater variation explained by chromosomes 1 (~threefold increase in AA), 4 (>40% increase in both EA and AA), 10 (>60% increase in EA) and 17 (over 60% increase in EA). For DBP, the LTA measurements are smaller than those from first visit values in EA (*r*_*cor*_ ~ 0.54, *P* = 9.4 x10^-3^) with the majority of the variation explained by chromosomes 2, 6, 10 and 11 (~ 50% to 75% decrease). In contrast, in AA, the overall variance explained by DBP-LTA is higher than first visit values (*r*_*cor*_ ~ 0.65, *P* = 9.4 x10^-4^), with the majority of the variation explained by chromosomes 1, 3 and 12 (~ 30% to 75% increase). Although these estimates have large standard errors, the overall heritability estimates are roughly similar between first visit and LTA measures, yet the chromosomal pattern of the variance explained differ considerably. In other words, issues beyond precision may modulate what we observe for LTA versus V1 heritability [25,26].

One expects that the whole-genome estimate of heritability is the sum of the chromosome-specific estimates unless there is correlation among the chromosomes. In our analyses, the chromosome sum and whole genome estimates are close to identical at 20% (EA)/50% (AA) for SBP and 27%(EA)/39%(AA) for DBP (Table 2). Thus, BP variation is additive and arises from the individual contributions of numerous polymorphisms. If genetic effects are approximately equal for all contributory alleles then the variation in contribution of these loci is highly dependent on their frequency, being proportional to heterozygosity. Therefore, we analyzed the variance contributions by minor allele frequency (MAF) class by binning each allele into five equal frequency classes between 0 and 50%, using first visit SBP and DBP (Table 3). Given the standard errors of the heritability estimates, the general conclusion is that the variance explained, for both SBP and DBP, is roughly equivalent for all MAF classes with minor differences. The estimated SBP genetic variance for the five MAF categories ranged from 0.025 to 0.056 (se 0.020–0.029) in EA, and from 0.023 to 0.24 (se 0.074–0.156) in AA, respectively, with the highest proportion being from SNPs with MAFs 0.1–0.2 in EA and MAFs 0.0–0.1 in AA. The estimated DBP genetic variance for the five MAF categories ranged from 0.016 to 0.068 (se 0.019–0.031) in EA, and from 0.000 to 0.104 (se 0.073–0.152) in AA, respectively, with the highest proportion being from SNPs with MAFs 0.1–0.2 in EA and MAFs 0.0–0.3 in AA. Once again, all estimates appear to be additive at the genome level. Thus, SBP and DBP behave analogously but with some small, and non-significant, population differences: clearly, BP heritability appears to be contributed by alleles of various frequencies across the spectrum. The more remarkable feature is the rough equivalency of heritability by MAF class despite there being a greater number of polymorphisms as MAF decreases and the consequent expectation that there are larger numbers of causal alleles at lower MAFs and that lower frequency alleles have larger effect[19].

**Fig 2:**
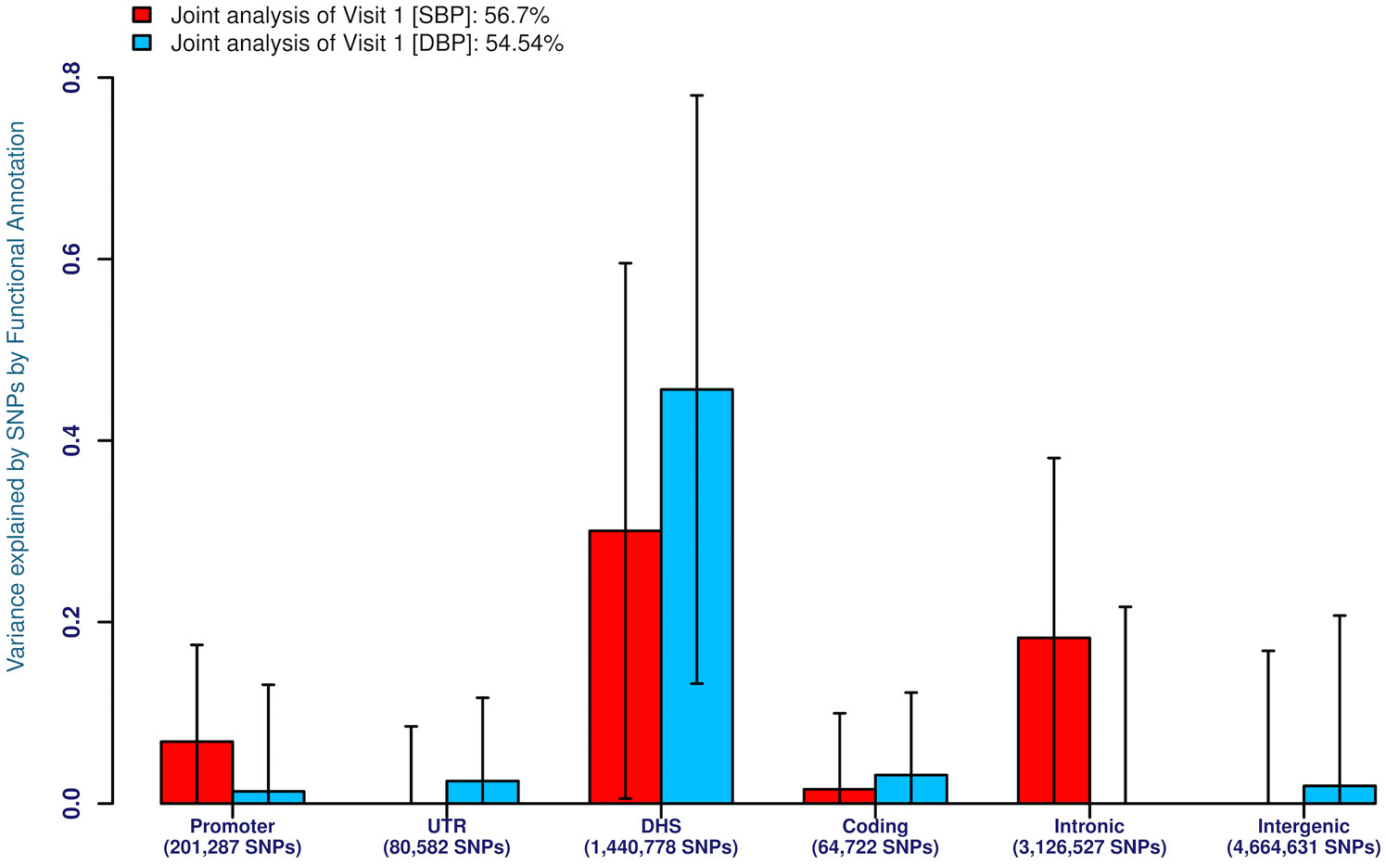
**(a)** Estimates of the variance explained for SBP (red) and DBP (blue) by functional annotation class (promoter, UTR, DHS, exon, intron, intergenic) by joint analysis using genotyped and imputed SNPs for first-visit SBP and DBP respectively in 8,901 EA individuals, **(b)** Legend as in **(a)** but for 2,860 AA individuals.

We next investigated the likely locations of these common alleles modulating BP variation: were they preferentially located within genes or in non-coding genomic regions? To do so we classified all genotyped and imputed SNPs by their location within an annotated promoter, DNase I hypersensitive site (DHS), coding exon, intron or UTR (Fig 2). We performed analyses on both first visit and LTA BP values but only first visit measures are shown given the similarity of the results. Strikingly, in EA, irrespective of whether SBP or DBP is considered, almost the entire heritability contribution is from DHSs (Fig 2a). On the other hand, in AA, SBP and DBP give different results (Fig 2b). For SBP, the heritability is relatively equally contributed by DHSs and intronic sites with small contributions from coding and promoter SNPs. For DBP, however, almost the entire heritability contribution is from DHSs. These results suggest basic population-level EA versus AA differences in the genetic causes of BP heritability that need to be assessed in future studies since the modest sample sizes here may lead to sampling variation.

**Fig 3:**
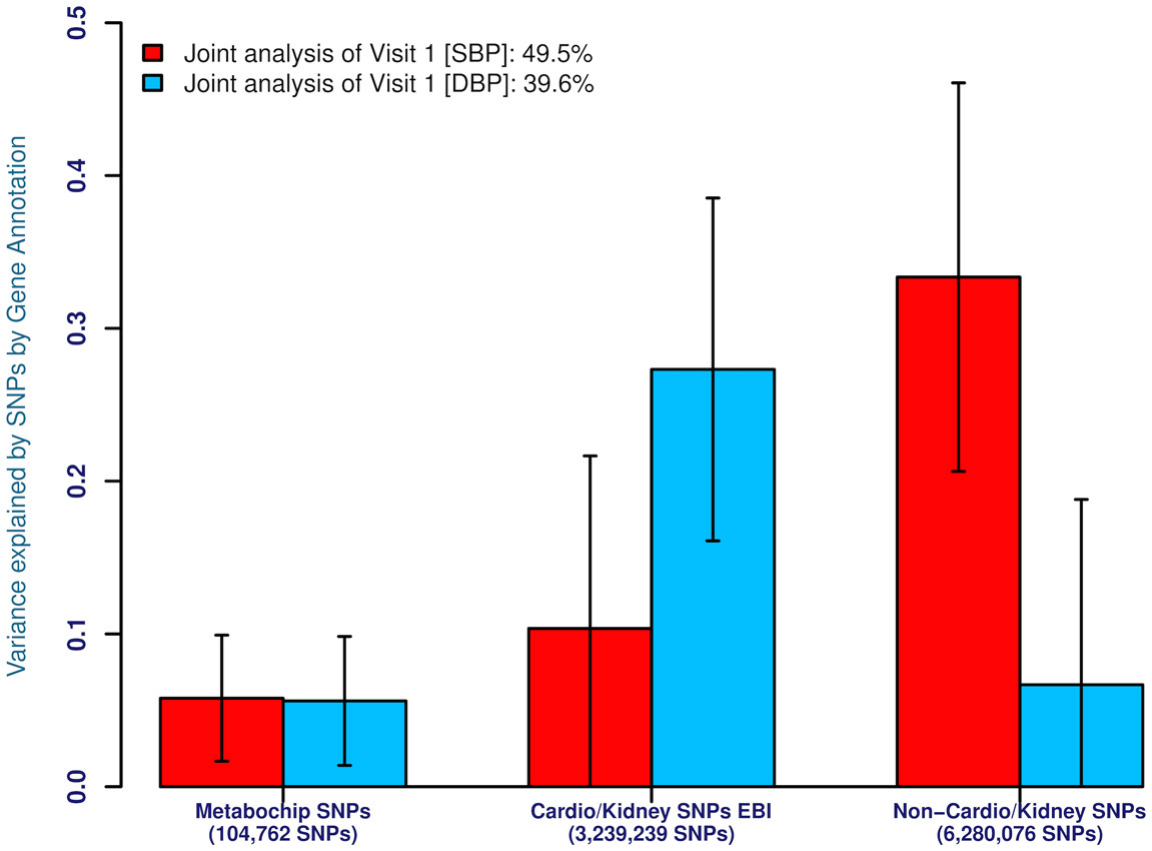
**(a)** Proportion of the genetic variance explained by SNPs for SBP and DBP for genotyped and imputed SNPs within known gene annotations (Metabochip SNPs = Markers associated with metabolic traits; Cardio/Kidney SNPs EBI = Markers associated with cardiovascular traits annotated by EBI; Non-Cardio/Kidney SNPs = Markers with no known association with cardiovascular or renal tissue) in 8,901 EA individuals. The trait analyzed is first-visit SBP and DBP with analyses for genotyped and imputed SNPs, **(b)** Legend as in **(a)** but for 2,860 AA individuals.

A second functional annotation that we used was the expression site of the gene closest to the SNP being classified (Fig 3a and 3b). Given current hypotheses about BP etiology, we expect that known cardiovascular- and renal-related genes should explain a significant fraction of BP heritability. We used the EBI resource of BHF-UCL[27] and KRUK[28] to annotate ~5,000 cardiovascular and renal genes within which we had 2,1721,073 and 3,239,239 genotyped and imputed SNPs in EA and AA, respectively. These variants were further annotated as to their membership on the MetaboChip array, a custom-designed reagent that enriched for variants known to be associated with any cardiovascular and metabolic trait/disease, including prior BP markers [29]. These comprised 100,946 and 104,762 genotyped and imputed SNPs in EA and AA samples, respectively (termed Metabochip SNPs). The remainders of the genes were annotated as non-cardiovascular-and-renal-related (~16,000 genes) within which we had 4,136,236 and 6,280,076 genotyped and imputed SNPs in EA and AA, respectively. Once again, we performed analyses on both first visit and LTA BP values but only first visit measures are shown given the similarity of the results. Surprisingly, in EA, irrespective of whether SBP or DBP is considered, the majority of the heritability arises from loci that are not cardiovascular or renal (Fig 3a). A large proportion of the variance (35%) is tagged by Cardio-Metabochip markers for both SBP and DBP, despite them accounting for less than 1.5% of the number of genotyped and imputed SNPs: no genetic variance is tagged by cardiovascular and renal genes for SBP while a moderate proportion of the heritability is detected for DBP (20%). On the other hand, in AA, SBP and DBP give different results (Fig 3b). For SBP, the heritability is predominantly promoted by genes that are not cardiovascular or renal, with small contributions from the Metabochip markers (15%) and loci harboring cardiovascular and renal genes (20%). For DBP, however, the majority of the heritability contribution arises from loci harboring cardiovascular and renal genes; whereas a small contribution of the heritability is captured by Metabochip markers (10%) and loci harboring non cardiovascular-and-renal-related genes (10%).

## Discussion

There is still considerable debate as to the relative importance of common versus rare variants in the inter-individual variation of complex traits[16]. Contemporary statistical methods now allow a direct estimate of the heritability from genome-wide marker data from unrelated phenotyped individuals[18,19]. These estimates can be compared to the classical estimates obtained from family and twin data, and these methods also allow heritability estimates from any subset of the genome to test genetic and etiological hypotheses. In this study, we explored this question for SBP and DBP by identifying the extent to which common variants can explain the amounts and distribution of SBP and DBP variation within the genome and with respect to allele frequency, coding versus non-coding DNA and sites of gene expression. We used single nucleotide polymorphism (SNP) data from the ARIC (Atherosclerosis Risk in Communities) population-based cohort study to demonstrate that the heritability attributable to additive effects of common variants assayed in this study for SBP and DBP is 20% (EA)/50% (AA) and 27% (EA)/39% (AA), respectively (Table 2 and Table 3). These estimates are not dependent on the number of SNPs used (i.e. imputation of genotyped SNPs increased the numbers of markers but still include those that were highly correlated to the primary genotyped set of common alleles) or whether we used a more stringent definition of unrelated individuals in the estimation (S3 and S4 Tables). These estimates compare favorably to estimates obtained from family[30] and twin studies[31] of adults that vary between 42% and 39–40% for SBP and DBP, respectively (S5 Table). One aspect to note is that these genome-wide heritability estimates appear to be accurate and have coefficients of variation of <15%.

The BP heritability estimates provided here strongly assert that the majority of inter-individual variation in BP detected today can be attributed to polymorphisms and not rare alleles, since the directly genotyped SNPs used had 1% or greater allele frequency. Genome-wide association studies, to date, have identified ~50 BP loci with a combined effect of ~2% of the phenotypic variance from common alleles with frequency ~30% on average [4–12]. It is well known that current GWAS are underpowered and that many BP loci remain undetected after stringent control for statistical significance. Our results suggest that the vast majority of these causal factors are indeed common (polymorphic) and remain undetected since their frequencies are in the 1–30% range. Consequently, the gap of undetected loci is likely very large. We have previously indicated that the typical SBP/DBP allelic effect is ~0.05σ (where σ^2^ is the phenotypic variance) so that the variance explained per SNP is 2pq(0.05σ)^2^ or 0.00105 (0.105%) where p ~ 30% is the (average) minor allele frequency. If this value were typical then our observed 25% heritability in this study can be explained through ~240 common variants at independent loci. We suspect the actual number to be far larger because there is both a statistical distribution of allelic effects, many likely smaller that we detect here, and most alleles will have lower frequencies. Assuming that 1000 or more BP loci lead to inter-individual phenotypic variation would not be exceptional and identifying them will involve GWAS in much larger sample sizes.

The analysis here sheds light on some of the properties of these putative causal BP alleles. First, the additive chromosomal-level and the joint genome-level analyses provide near identical estimates suggesting that BP alleles are additive in genetic action (S1–S4 Tables). The numbers of such factors are generally proportional to chromosome size although some individual chromosomes harbor a surfeit of BP loci (e.g. chromosome 2[32]), while others are impoverished (Fig 1). The reasons for such spatial clustering are unknown and will be important to unravel. Second, the vast majority of these causal alleles reside in non-coding DNA, within introns and inter-genic DNA at DHSs (Fig 3). There is increasing recognition that most of these changes are at cis-regulatory elements modulating local gene expression [33,34]. Third, causal alleles are widely distributed throughout the allele frequency spectrum with no particular tendency of an enrichment of rare allele contributors to BP. Fourth, functional annotation, with respect to association with cardiovascular and renal genes or SNPs previously associated with cardiovascular and metabolic diseases showed considerable enrichment. Nevertheless, there were distinct population differences. Fifth, LTA measurements, expected to describe a more accurate estimate of an individual’s long-term BP value, made only limited difference to the variance captured. These conclusions are quantitatively an underestimate of the additive genomic influence because they are limited to SNPs with a minor allele frequency beyond 1% and other SNPs reliably (R^2^ > 0.3) imputed from them; rarer variants and not well-imputed SNPs are, therefore, excluded. Also, causal SNPs that were not highly correlated with the SNPs on the genotyping array or after imputation were also missed.

The above conclusions need to be replicated in independent studies to assess how robust these conclusions are. Nevertheless, the main challenge in complex trait genetics remains the specific identification of the causal non-coding alleles, and the genes they affect, underlying SBP and DBP variability. Although GWASs are increasing in sample size and identifying a greater number of loci it is unlikely that they can achieve saturation identification by statistical detection alone. Our analyses suggest that the vast majority of these alleles are common, distributed across the genome, non-coding and not associated with known cardiovascular or renal genes [35]. Thus, we propose that alternate strategies be also considered. One such strategy might be the partitioning of the genome and its variation into the two following ‘functional’ segments, the ~5,000 cardiovascular and renal gene coding sequences and all DHSs. Searches for genetic variation, either by the analyses methods here or by direct GWAS, affecting SBP and DBP within these segments may lead to the identification of a larger number of causal factors.

In conclusion, we estimated additive genetic variation that is captured by genotyped and imputed SNPs for BP, and partitioned this variation according to chromosome, allele frequency, and gene annotation. We provide compelling evidence that a substantial proportion of variance for blood pressure trait is explained by common SNPs, and thereby, give insights into the genetic architecture of BP trait. However, it is likely that variants other than the ones considered here and those with small effect need to be considered in addition to common SNPs.

## Materials and Methods

Phenotype data were available for 15,792 participants from the ARIC (Atherosclerosis Risk in Communities) study, which is a population-based, prospective, epidemiologic study of cardiovascular disease in European ancestry (EA) and African ancestry (AA) volunteers aged 45–64 years at baseline (1987–89), conducted in four US communities [36]. All ARIC subjects provided written, informed consent to participate in research protocols that were approved by the University of North Carolina at Chapel Hill, Chapel Hill, NC institutional review board. Only de-identified data was used for these analyses. This analysis is focused on both EA and AA participants. Cohort members completed up to four clinic examinations between 1987 and 1998 that were conducted approximately three years apart. Clinical examinations for ARIC participants assessed cardiovascular risk factors and diet, undertook various clinical and laboratory measurements, and measured numerous social variables such as education and income. Genome-wide SNP genotypes in 9,747 self-identified EA and 3,207 self-identified AA subjects were obtained using the Affymetrix Gene Chip Human Mapping Array Set 6.0. The genotype data were used to exclude some samples from analyses for the following reasons: 1) discordance with previous genotype data (n = 171 in EA; n = 11 in AA), 2) mismatch between genotype- and phenotype-based gender (n = 12 in EA), 3) previously unrecognized but suspected first or second degree relative of another participant (n = 355 in EA), 4) genetic outlier as assessed by average Identity by State (IBS) statistics (>8 standard deviations along any of first 10 principal components in EIGENSTRAT with 5 iterations using PLINK) (n = 308 in EA; n = 336 in AA). This led to an exclusion of 846 EA and 347 AA participants, resulting in a retained dataset of 8,901 and 2,860 unrelated EA and AA subjects, respectively. In parallel, to check if shared environmental effects and/or causal variants not captured could further bias our variance estimates, we also tested a more stringent cut-off, after estimation of the pairwise genetic relationship using all autosomal markers, by excluding one of each pair of individuals with an estimated genetic relatedness of >0.025 (kinship less than 2^nd^ cousins). This led to an exclusion of 2,833 unrelated EA and 1,123 unrelated AA subjects, respectively, resulting in a retained dataset of correspondingly 6,914 and 1,737 genetically “unrelated” EA and AA participants [20].

Blood pressures were measured by a random zero mercury sphygmomanometer following a standard protocol described elsewhere [37]. The phenotypes used for this analysis were SBP, DBP at the first examination (visit). Subjects under antihypertensive treatments were adjusted for potential medication effects by adding 10 and 5 mm Hg to observed SBP and DBP measurements, respectively. Hypertensive participants were defined as either having SBP ≥ 140 mm Hg or DBP ≥ 90 mmHg or using an antihypertensive drug at the time of examination. We fit regression models for SBP and DBP separately, after adjusting for the following covariates: sex, age, age squared and body mass index (BMI). The blood pressure traits analyzed here were the residuals from this regression.

To obtain long-term averaged (LTA) BP traits, we averaged repeated BP measurements for study participants; individuals with four repeated BP measures at least 1 year apart were included in our analyses. At each study visit, we fit a linear regression model by using covariate adjustment in a manner identical to what was done for first visit measures to generate visit-specific BP residuals. These residual values were subsequently averaged over all available visits, and the final averaged residual was the LTA trait analyzed (termed LTA SBP, LTA DBP).

Quality control on genotypes has been described elsewhere [38]. Nevertheless, after pruning 308 EA and 334 AA individual participants from the raw data as genetic outliers, we performed imputation in EA and AA subjects to the 1000 Genomes reference panel. Imputation in EA participants was performed using a hidden Markov model as implemented in the software packages MaCH (v1.0.16) and Minimac (v4.6) [39]. Each chromosome was first phased to estimate haplotypes using MaCH and then the phased haplotypes were used, along with the 1000G Interim Phase I Haplotype reference panel, resulting in >37 million SNPs for imputation using Minimac. Imputation in AA participants was completed with IMPUTE version 2 (v2.1.2) and involved a two-step procedure for each chromosome: phasing to generate haplotype followed by imputation using similar reference panels. SNPs used for imputation were restricted to those with the following features: MAF >0.5%, missing data per SNP < 5%, and Hardy-Weinberg equilibrium (HWE) P >10^-5^. Of the 839,048 genotyped markers, 656,362 and 772,638 genotyped autosomal SNPs in EA and AA, respectively, passed the initial quality filters and were used for imputation. In this study, sex chromosomes were excluded from the analysis of blood pressure.

Descriptive statistics and regression of the phenotype on age, age-squared, sex, BMI and PCs, were carried out using R version 2.6.0 (The R Foundation for Statistical Computing). The statistical method utilized here is detailed in Yang *et al*[18]. Briefly, a genetic relationship matrix (GRM) for each pair of individuals was calculated as the sum of the products of SNP coefficients between two individuals scaled by the SNP heterozygosity for all genotyped and imputed SNPs across the genome. Subsequently, the GRM was used in a linear mixed model to estimate the variance captured by all the autosomal SNPs via restricted maximum likelihood analysis. This was expressed as a linear function of the total amount of the additive effects due to SNPs associated with causal markers and residual effects: $y\;=\;X\beta +\sum_{i\;=\;1}^{p}V_{A_{i}}+\;\varepsilon \;$ where ***y*** is an **N × 1** vector of systolic or diastolic blood pressure measurements with **N** being the sample size, ***β*** a vector for fixed effects such as sex, age, age squared and BMI, ***X*** the genotype incidence matrix relating to individuals, ***V***_***A***_ a vector of random additive genetic effects partitioned on aggregate of all autosomal SNPs estimated from whole-genome markers (***p*** = 1; $y\;=\;X\beta +\sum_{i\;=\;1}^{1}V_{A_{i}}+\;\varepsilon$). The proportion of variance explained by whole-genome markers is the narrow-sense heritability, i.e., $h^{2}\;=\;\sigma_{A}^{2}/\;\sigma_{P}^{2}$ where $\sigma_{P}^{2}$ is the total phenotypic variance.

A pairwise genomic relationship matrix (GRM) was estimated using the high quality (call rate > 95%; MAF ≥ 1%; Hardy-Weinberg Equilibrium (HWE) P > 10^-6^) autosomal genotypes at 6,365,596 SNPs for EA and 9,578,527 SNPs for AA subjects, including all those directly genotyped and those imputed markers with MAF ≥ 1% (imputation R^2^ ≥ 0.3). To avoid phenotypic resemblance due to non-additive genetic effects and common environmental influences, we excluded one of each pair of individuals with an estimated genetic relationship >0.025 (i.e., equivalent to 2^nd^ cousins or closer). Consequently, we created a second dataset that included only 6,914 and 1,737 genetically “unrelated” EA and AA study participants, respectively, to assess whether they impacted our conclusions.

The variance estimate from the entire genome can also be partitioned into non-overlapping subsets of SNPs defined by any specific criteria: if ***p*** such classes are considered then $var(V_{A})=\;\sum_{i=1}^{p}M_{A_{i}}+\;\sigma_{A}^{2}$ where **M**_***A***_ is the GRM estimated from the whole-genome genotyped or imputed markers, $\sigma_{A}^{2}$ is the variance explained by all SNPs, and ***ε*** is a vector of random error effects. These GRMs were fit in a mixed linear model (MLM) to SBP and DBP and restricted maximum likelihood (REML) methods were used to estimate the proportion of variance explained by any predefined set of genetic markers. Two types of analyses were performed that estimated the proportion of variance explained by the sum of the effects from individual chromosomes and by the whole-genome: for the first analysis, we fit 22 pairwise relationship matrices simultaneously (termed, joint analysis) while in the second we merged these relationship matrices into one GRM (termed, combined analysis). The specific partitions we considered were chromosomal number (***p*** = 22; autosomes), minor allele frequency (MAF) class (***p*** = 5; 0–50% in 10% intervals), specific functional genomic annotation of SNPs developed by *Gusev et al*. [40] (***p*** = 6; promoter, DHS, UTRs, coding, intronic, intergenic), functional annotation based on gene expression in known cardiovascular or renal associated-genes [41] and genes annotated as Cardiovascular and Renal by EMBL-EBI [28,42] and SNPs from the Metabochip array[29]. Finally, the variance estimate from the entire genome was partitioned by blood pressure loci from the NHGRI GWAS catalog [43] (http://www.genome.gov/gwastudies) and the literature (3,5,6,7,8,10,11,12,13,14) and including all markers in strong LD (r^2^ ≥ 0.8) with the sentinel SNP using SNAP [44].

## Supporting Information

S1 Table Proportion of the genetic variance explained by each chromosome and the whole genome using 8,901 EA individuals. (DOCX)

S2 Table Proportion of the genetic variance explained by each chromosome and the whole genome using 2,860 AA individuals. (DOCX)

S3 Table Proportion of the genetic variance explained by each chromosome and the whole genome using 6,914 EA unrelated individuals. (DOCX)

S4 Table Proportion of the genetic variance explained by each chromosome and the whole genome using 1,763 AA unrelated individuals. (DOCX)

S5 Table Estimates of the SBP and DBP heritability from classical family and twin studies. (DOCX)
