# Supplementary material for: Direct Estimates of the Genomic Contributions to Blood Pressure Heritability within a Population-Based Cohort (ARIC)
Source: PLoS One. 2015 Jul 10;10(7):e0133031. doi: 10.1371/journal.pone.0133031 (PMC4498745; doi:10.1371/journal.pone.0133031)
Supplement: S1 Table — (DOCX) [file pone.0133031.s001.docx]

**S1 Table.** Proportion of the genetic variance explained by each chromosome and the whole genome using 8,901 EA individuals.

| **EA** | | **SBP** | | **DBP** | |
| --- | --- | --- | --- | --- | --- |
| **SNPs** | | **V1 (N=8,901)** | **LTA (N=8,474)** | **V1 (N=8,901)** | **LTA (N=8,474)** |
| **Chr** | ***LC (Mb)*** | ***h^2^ ± s.e.*** | ***h^2^ ± s.e.*** | ***h^2^ ± s.e.*** | ***h^2^ ± s.e.*** |
| 1 | 249.25 | 0.0101 ± 0.0105 | 0.0044 ± 0.0106 | 0.0073 ± 0.0104 | 0.0103 ± 0.0108 |
| 2 | 243.2 | 0.0257 ± 0.0112 | 0.0223 ± 0.0116 | 0.0231 ± 0.0112 | 0.0086 ± 0.0114 |
| 3 | 198.02 | 0.009 ± 0.0093 | 0.019 ± 0.0099 | 0.0181 ± 0.0095 | 0.0261 ± 0.0101 |
| 4 | 191.15 | 0.0221 ± 0.0103 | 0.035 ± 0.0111 | 0.022 ± 0.0102 | 0.0254 ± 0.0107 |
| 5 | 180.92 | 0.0115 ± 0.0092 | 0.0087 ± 0.0095 | 0.0102 ± 0.0091 | 0.0175 ± 0.01 |
| 6 | 171.12 | 0 ± 0.0083 | 0 ± 0.0084 | 0.0179 ± 0.0088 | 0.0041 ± 0.0081 |
| 7 | 159.14 | 0 ± 0.0085 | 0 ± 0.0092 | 0.0218 ± 0.0098 | 0.0129 ± 0.0098 |
| 8 | 146.36 | 0.0103 ± 0.0081 | 0.0137 ± 0.0087 | 0.0081 ± 0.0082 | 0.0083 ± 0.0084 |
| 9 | 141.21 | 0.0109 ± 0.0084 | 0.0142 ± 0.009 | 0.0041 ± 0.0081 | 0.0034 ± 0.0084 |
| 10 | 135.53 | 0.0104 ± 0.0089 | 0.0252 ± 0.0102 | 0.0137 ± 0.0089 | 0.0012 ± 0.0084 |
| 11 | 135.01 | 0.0175 ± 0.0082 | 0.0168 ± 0.0085 | 0.0262 ± 0.0091 | 0.0175 ± 0.009 |
| 12 | 133.85 | 0.0208 ± 0.0092 | 0.0211 ± 0.0096 | 0.019 ± 0.0089 | 0.0233 ± 0.0095 |
| 13 | 115.17 | 0.0118 ± 0.0076 | 0.0023 ± 0.0072 | 0.0078 ± 0.0073 | 0.0017 ± 0.007 |
| 14 | 107.35 | 0.0137 ± 0.0074 | 0.0139 ± 0.0077 | 0 ± 0.0066 | 0 ± 0.0066 |
| 15 | 102.53 | 0.0113 ± 0.0073 | 0.0032 ± 0.0069 | 0.012 ± 0.0072 | 0.0201 ± 0.008 |
| 16 | 90.35 | 0.0033 ± 0.0074 | 0.0058 ± 0.0075 | 0.0221 ± 0.0083 | 0.0177 ± 0.0083 |
| 17 | 81.2 | 0.005 ± 0.0058 | 0.0177 ± 0.0076 | 0.0059 ± 0.006 | 0.0084 ± 0.0069 |
| 18 | 78.08 | 0.0012 ± 0.0066 | 0.0036 ± 0.0072 | 0.0001 ± 0.0065 | 0.0051 ± 0.0069 |
| 19 | 59.13 | 0 ± 0.0044 | 0 ± 0.0044 | 0.0035 ± 0.0048 | 0.0098 ± 0.0058 |
| 20 | 63.03 | 0.0024 ± 0.0063 | 0.0034 ± 0.0066 | 0.0086 ± 0.0068 | 0.0046 ± 0.0069 |
| 21 | 48.13 | 0.0064 ± 0.0053 | 0.0047 ± 0.0054 | 0.0123 ± 0.0057 | 0.0096 ± 0.0057 |
| 22 | 51.3 | 0.0028 ± 0.005 | 0 ± 0.0049 | 0.0115 ± 0.0056 | 0.0131 ± 0.006 |
| **Total** | 2,881.03 | ***0.206*** | ***0.2349*** | ***0.2752*** | ***0.248*** |
| **Combined** | | ***0.201 ± 0.035*** | ***0.227 ± 0.037*** | ***0.2642 ± 0.035*** | ***0.233 ± 0.038*** |
| **P** | | ***3.16x10^-10^*** | ***1.32x10^-10^*** | ***5.2x10^-11^*** | ***8.37x10^-11^*** |
