# Supplementary material for: Direct Estimates of the Genomic Contributions to Blood Pressure Heritability within a Population-Based Cohort (ARIC)
Source: PLoS One. 2015 Jul 10;10(7):e0133031. doi: 10.1371/journal.pone.0133031 (PMC4498745; doi:10.1371/journal.pone.0133031)
Supplement: S2 Table — (DOCX) [file pone.0133031.s002.docx]

**S2 Table.** Proportion of the genetic variance explained by each chromosome and the whole genome using 2,860 AA individuals.

| **AA** | | **SBP** | | **DBP** | |
| --- | --- | --- | --- | --- | --- |
| **SNPs** | | **V1 (N=2,860)** | **LTA (N=2,749)** | **V1 (N=2,860)** | **LTA (N=2,749)** |
| **Chr** | ***LC (Mb)*** | ***h^2^ ± s.e.*** | ***h^2^ ± s.e.*** | ***h^2^ ± s.e.*** | ***h^2^ ± s.e.*** |
| 1 | 249.25 | 0.0261 ± 0.0381 | 0.0786 ± 0.0486 | 0.0041 ± 0.0358 | 0.0562 ± 0.048 |
| 2 | 243.2 | 0.1305 ± 0.0514 | 0.0842 ± 0.0509 | 0.042 ± 0.0488 | 0.0412 ± 0.0492 |
| 3 | 198.02 | 0 ± 0.0391 | 0 ± 0.0394 | 0.039 ± 0.038 | 0.0735 ± 0.0412 |
| 4 | 191.15 | 0.0166 ± 0.0419 | 0.0577 ± 0.0462 | 0 ± 0.0448 | 0.0009 ± 0.0423 |
| 5 | 180.92 | 0.0717 ± 0.0453 | 0.0856 ± 0.0472 | 0.0874 ± 0.045 | 0.0634 ± 0.0429 |
| 6 | 171.12 | 0 ± 0.037 | 0 ± 0.0357 | 0 ± 0.0365 | 0.0192 ± 0.0368 |
| 7 | 159.14 | 0.0306 ± 0.0377 | 0.0426 ± 0.04 | 0 ± 0.0359 | 0.042 ± 0.0402 |
| 8 | 146.36 | 0.0539 ± 0.0384 | 0.0154 ± 0.0326 | 0.0361 ± 0.0376 | 0.0184 ± 0.0362 |
| 9 | 141.21 | 0.0186 ± 0.0343 | 0.0304 ± 0.0363 | 0.0102 ± 0.033 | 0.0042 ± 0.0308 |
| 10 | 135.53 | 0.0195 ± 0.0375 | 0 ± 0.036 | 0.0364 ± 0.0379 | 0.0216 ± 0.0349 |
| 11 | 135.01 | 0.0645 ± 0.0342 | 0.0726 ± 0.0367 | 0.0517 ± 0.0334 | 0.015 ± 0.0288 |
| 12 | 133.85 | 0.0214 ± 0.0314 | 0.0285 ± 0.0332 | 0.0048 ± 0.0266 | 0.0566 ± 0.0367 |
| 13 | 115.17 | 0 ± 0.0317 | 0.0174 ± 0.0334 | 0.0359 ± 0.0344 | 0.0371 ± 0.0345 |
| 14 | 107.35 | 0 ± 0.0282 | 0 ± 0.0253 | 0 ± 0.0291 | 0 ± 0.0271 |
| 15 | 102.53 | 0.0048 ± 0.0238 | 0.0181 ± 0.0299 | 0.0059 ± 0.0263 | 0 ± 0.0295 |
| 16 | 90.35 | 0.0042 ± 0.0316 | 0 ± 0.0321 | 0 ± 0.0303 | 0 ± 0.0318 |
| 17 | 81.2 | 0.0034 ± 0.0256 | 0 ± 0.0237 | 0.0066 ± 0.0242 | 0 ± 0.0162 |
| 18 | 78.08 | 0 ± 0.03 | 0.0038 ± 0.0321 | 0.0046 ± 0.0303 | 0.033 ± 0.0337 |
| 19 | 59.13 | 0.027 ± 0.021 | 0.0254 ± 0.0204 | 0.0076 ± 0.0178 | 0.0271 ± 0.0208 |
| 20 | 63.03 | 0.0101 ± 0.0257 | 0.0046 ± 0.0258 | 0.012 ± 0.0257 | 0 ± 0.0258 |
| 21 | 48.13 | 0 ± 0.0159 | 0.0014 ± 0.0156 | 0 ± 0.0195 | 0 ± 0.0191 |
| 22 | 51.3 | 0.0016 ± 0.0211 | 0.0075 ± 0.0215 | 0.0061 ± 0.0202 | 0.0032 ± 0.0221 |
| **Total** | 2,881.03 | ***0.504*** | ***0.574*** | ***0.39*** | ***0.512*** |
| **Combined** | | ***0.498 ± 0.126*** | ***0.571 ± 0.131*** | ***0.374 ± 0.122*** | ***0.55 ± 0.132*** |
| **P** | | ***7.9x10^-6^*** | ***7.78x10^-7^*** | ***3.9x10^-5^*** | ***3.2x10^-7^*** |
