# Supplementary material for: Direct Estimates of the Genomic Contributions to Blood Pressure Heritability within a Population-Based Cohort (ARIC)
Source: PLoS One. 2015 Jul 10;10(7):e0133031. doi: 10.1371/journal.pone.0133031 (PMC4498745; doi:10.1371/journal.pone.0133031)
Supplement: S3 Table — (DOCX) [file pone.0133031.s003.docx]

**S3 Table.** Proportion of the genetic variance explained by each chromosome and the whole genome using 6,914 EA

unrelated individuals.

| **EA** | | **SBP** | | **DBP** | |
| --- | --- | --- | --- | --- | --- |
| **SNPs** | | **V1 (N=6,914)** | **LTA (N=6,591)** | **V1 (N=6,914)** | **LTA (N=6,591)** |
| **Chr** | ***LC (Mb)*** | ***h^2^ ± s.e.*** | ***h^2^ ± s.e.*** | ***h^2^ ± s.e.*** | ***h^2^ ± s.e.*** |
| 1 | 249.25 | 0.0097 ± 0.0132 | 0 ± 0.0135 | 0.0005 ± 0.0128 | 0.0048 ± 0.0134 |
| 2 | 243.2 | 0.041 ± 0.015 | 0.025 ± 0.0152 | 0.0315 ± 0.0147 | 0.0094 ± 0.0147 |
| 3 | 198.02 | 0.0068 ± 0.0117 | 0.0145 ± 0.0122 | 0.0175 ± 0.012 | 0.0209 ± 0.0125 |
| 4 | 191.15 | 0.0315 ± 0.0135 | 0.0449 ± 0.0144 | 0.0463 ± 0.014 | 0.0377 ± 0.014 |
| 5 | 180.92 | 0.0074 ± 0.0112 | 0.0178 ± 0.0124 | 0 ± 0.0111 | 0.0031 ± 0.0118 |
| 6 | 171.12 | 0 ± 0.011 | 0 ± 0.011 | 0.0119 ± 0.011 | 0 ± 0.0099 |
| 7 | 159.14 | 0.0062 ± 0.0112 | 0 ± 0.0116 | 0.008 ± 0.0116 | 0 ± 0.0115 |
| 8 | 146.36 | 0.0158 ± 0.0108 | 0.0168 ± 0.0117 | 0.0088 ± 0.0105 | 0.0034 ± 0.0104 |
| 9 | 141.21 | 0.0103 ± 0.0108 | 0.008 ± 0.011 | 0.0037 ± 0.0103 | 0.0024 ± 0.0106 |
| 10 | 135.53 | 0.0006 ± 0.0106 | 0.0134 ± 0.0118 | 0.02 ± 0.0117 | 0.0101 ± 0.0113 |
| 11 | 135.01 | 0.0075 ± 0.0083 | 0.0036 ± 0.0084 | 0.0121 ± 0.01 | 0.01 ± 0.0106 |
| 12 | 133.85 | 0.0287 ± 0.0121 | 0.0321 ± 0.013 | 0.0194 ± 0.0111 | 0.0317 ± 0.0125 |
| 13 | 115.17 | 0.01 ± 0.0095 | 0.0016 ± 0.0094 | 0.0161 ± 0.0098 | 0 ± 0.0088 |
| 14 | 107.35 | 0.0092 ± 0.009 | 0.0031 ± 0.0087 | 0 ± 0.0087 | 0 ± 0.0086 |
| 15 | 102.53 | 0.0051 ± 0.0085 | 0 ± 0.0085 | 0.0116 ± 0.009 | 0.0191 ± 0.0099 |
| 16 | 90.35 | 0.0008 ± 0.0092 | 0.0009 ± 0.0096 | 0.0242 ± 0.0105 | 0.0189 ± 0.0104 |
| 17 | 81.2 | 0.0056 ± 0.0074 | 0.0089 ± 0.0086 | 0.0078 ± 0.0079 | 0.003 ± 0.0077 |
| 18 | 78.08 | 0 ± 0.0086 | 0 ± 0.0087 | 0 ± 0.0086 | 0.0008 ± 0.0087 |
| 19 | 59.13 | 0 ± 0.0057 | 0 ± 0.006 | 0.004 ± 0.0064 | 0.0088 ± 0.007 |
| 20 | 63.03 | 0.0006 ± 0.0078 | 0.0004 ± 0.0081 | 0.0057 ± 0.0081 | 0.0021 ± 0.0085 |
| 21 | 48.13 | 0.009 ± 0.007 | 0.0067 ± 0.007 | 0.0096 ± 0.007 | 0.0109 ± 0.0072 |
| 22 | 51.3 | 0.0041 ± 0.0064 | 0 ± 0.0063 | 0.0185 ± 0.0076 | 0.0212 ± 0.0079 |
| **Total** | 2,881.03 | ***0.21*** | ***0.197*** | ***0.277*** | ***0.218*** |
| **Combined** | | ***0.196 ± 0.046*** | ***0.186 ± 0.048*** | ***0.266 ± 0.045*** | ***0.204 ± 0.048*** |
| **P** | | ***2.32x10^-6^*** | ***4.62x10^-5^*** | ***4.1x10^-1^2*** | ***8.85x10^-6^*** |
