# Supplementary material for: Direct Estimates of the Genomic Contributions to Blood Pressure Heritability within a Population-Based Cohort (ARIC)
Source: PLoS One. 2015 Jul 10;10(7):e0133031. doi: 10.1371/journal.pone.0133031 (PMC4498745; doi:10.1371/journal.pone.0133031)
Supplement: S4 Table — (DOCX) [file pone.0133031.s004.docx]

**S4 Table.** Proportion of the genetic variance explained by each chromosome and the whole genome using 1,763 AA

unrelated individuals.

| **AA** | | **SBP** | | **DBP** | |
| --- | --- | --- | --- | --- | --- |
| **SNPs** | | **V1 (N=1,737)** | **LTA (N=1,670)** | **V1 (N=1,737)** | **LTA (N=1,670)** |
| **Chr** | ***LC (Mb)*** | ***h2 ± s.e.*** | ***h2 ± s.e.*** | ***h2 ± s.e.*** | ***h2 ± s.e.*** |
| 1 | 249.25 | 0 ± 0.0731 | 0.0446 ± 0.0818 | 0.0029 ± 0.0648 | 0.1518 ± 0.0845 |
| 2 | 243.2 | 0.1053 ± 0.087 | 0.0514 ± 0.0846 | 0.0275 ± 0.0843 | 0.1068 ± 0.0889 |
| 3 | 198.02 | 0 ± 0.07 | 0 ± 0.07 | 0.0681 ± 0.064 | 0.1034 ± 0.0673 |
| 4 | 191.15 | 0 ± 0.0734 | 0 ± 0.0621 | 0 ± 0.0683 | 0 ± 0.0697 |
| 5 | 180.92 | 0 ± 0.0658 | 0 ± 0.0744 | 0.0175 ± 0.0623 | 0.0291 ± 0.063 |
| 6 | 171.12 | 0 ± 0.0673 | 0 ± 0.0677 | 0 ± 0.0552 | 0 ± 0.0552 |
| 7 | 159.14 | 0 ± 0.0633 | 0.032 ± 0.0698 | 0 ± 0.0631 | 0.0058 ± 0.0637 |
| 8 | 146.36 | 0.0801 ± 0.0637 | 0.0339 ± 0.0599 | 0.0498 ± 0.0609 | 0.0009 ± 0.0527 |
| 9 | 141.21 | 0.0143 ± 0.0533 | 0.0257 ± 0.0582 | 0.0188 ± 0.0519 | 0.0162 ± 0.0494 |
| 10 | 135.53 | 0 ± 0.0625 | 0 ± 0.0662 | 0.0031 ± 0.0505 | 0.0033 ± 0.0555 |
| 11 | 135.01 | 0.0603 ± 0.0516 | 0.0279 ± 0.0494 | 0.0371 ± 0.0496 | 0.0239 ± 0.0509 |
| 12 | 133.85 | 0.0277 ± 0.0474 | 0.0185 ± 0.0486 | 0.0153 ± 0.0467 | 0.0314 ± 0.053 |
| 13 | 115.17 | 0.0139 ± 0.0522 | 0.0362 ± 0.0567 | 0.0137 ± 0.0535 | 0.0195 ± 0.0457 |
| 14 | 107.35 | 0.0229 ± 0.0444 | 0.0072 ± 0.0442 | 0.0036 ± 0.0419 | 0.0034 ± 0.0428 |
| 15 | 102.53 | 0.0188 ± 0.0412 | 0.0575 ± 0.0523 | 0.0197 ± 0.0419 | 0.0294 ± 0.0487 |
| 16 | 90.35 | 0 ± 0.04 | 0.0003 ± 0.0366 | 0 ± 0.0414 | 0 ± 0.0343 |
| 17 | 81.2 | 0 ± 0.0396 | 0 ± 0.0444 | 0 ± 0.0412 | 0 ± 0.038 |
| 18 | 78.08 | 0 ± 0.0466 | 0 ± 0.0488 | 0.0157 ± 0.0464 | 0.0224 ± 0.048 |
| 19 | 59.13 | 0.0029 ± 0.0282 | 0.0074 ± 0.0291 | 0.0535 ± 0.0351 | 0.0424 ± 0.0344 |
| 20 | 63.03 | 0.0027 ± 0.0458 | 0.0279 ± 0.0516 | 0.0082 ± 0.0475 | 0.0061 ± 0.0468 |
| 21 | 48.13 | 0.0069 ± 0.0321 | 0 ± 0.0291 | 0.0026 ± 0.026 | 0.0207 ± 0.0356 |
| 22 | 51.3 | 0.0124 ± 0.036 | 0.0016 ± 0.0335 | 0.0127 ± 0.0338 | 0 ± 0.0376 |
| **Total** | 2,881.03 | ***0.367*** | ***0.371*** | ***0.369*** | ***0.616*** |
| **Combined** | | ***0.409 ± 0.236*** | ***0.37 ± 0.236*** | ***0.378 ± 0.212*** | ***0.615 ± 0.238*** |
| **P** | | ***4.4x10^-2^*** | ***5.26x10^-2^*** | ***1.1x10^-2^*** | ***1.21x10^-3^*** |
