# Supplementary material for: Direct Estimates of the Genomic Contributions to Blood Pressure Heritability within a Population-Based Cohort (ARIC)
Source: PLoS One. 2015 Jul 10;10(7):e0133031. doi: 10.1371/journal.pone.0133031 (PMC4498745; doi:10.1371/journal.pone.0133031)
Supplement: S5 Table — (DOCX) [file pone.0133031.s005.docx]

**S5 Table.** Estimates of the SBP and DBP heritability from classical family and twin studies.

| **Trait** | **Sample Size** | **Heritability** | **Relatives** | **Study** | **Ref** |
| --- | --- | --- | --- | --- | --- |
|  |  |  |  |  |  |
| **SBP** | 1,585 | 0.42 | Family | FHS | 30 |
| **SBP** | 1,617 | 0.42 | Twins | Chinese Twins | 31 |
|  |  |  |  |  |  |
| **DBP** | 1,294 | 0.39 | Family | FHS | 30 |
| **DBP** | 1,617 | 0.4 | Twins | Chinese Twins | 31 |
